# Prevalence and Risk Factors of Asthma in Preschool Children in Shanghai, China: A Cross-Sectional Study

**DOI:** 10.3389/fped.2021.793452

**Published:** 2022-02-09

**Authors:** Jie Ren, Jing Xu, Pingbo Zhang, Yixiao Bao

**Affiliations:** ^1^Department of Respiratory Medicine, Shanghai Children's Medical Center, School of Medicine, Shanghai Jiao Tong University, Shanghai, China; ^2^Yangpu District Maternity and Child Healthcare Hospital, Shanghai, China; ^3^Shanghai Tonxin Clinic, Shanghai, China

**Keywords:** cross-sectional, risk factors, epidemiology, asthma, preschool

## Abstract

**Background:**

Previous studies have shown the increasing prevalence of childhood asthma around the world as well as in China. Nevertheless, little is known about the epidemiology of asthma in preschool children. Thus, the present study investigated the prevalence and severity of asthma in Shanghai, China, and identified related risk factors for asthma in children at the age of 3–6.

**Methods:**

Information was obtained through the International Study of Asthma and Allergies in Childhood (ISAAC) questionnaire. Risk factor analysis was carried out using univariate and multivariate logistic regression. The odds ratio (OR)/adjusted odds ratio (aOR) and the 95% confidence interval (CI) were determined.

**Results:**

A total of 6,183 children (3,165 boys and 3,018 girls) covering 12 communities were included in our study, with an average age of 4.2 ± 0.7 years. The prevalence of ever asthma, current asthma, and physician-diagnosed asthma was 16.0, 11.2, and 5.3%, respectively. Parental allergic history, including rhinitis and asthma, was significantly associated with asthma symptoms. The strongest association with current asthma was paternal asthma (aOR = 5.91, 95% CI 3.87–9.01), and maternal asthma had the second strongest association with current asthma (3.85; 2.40–6.17). Among personal factors, allergic rhinitis history, eczema history, food allergy history, and antibiotic use in the first year of life were significantly associated with current asthma (aOR = 1.89, 95% CI 1.52–2.34; aOR = 1.34, 95% CI 1.09–1.64; aOR = 1.68, 95% CI 1.37–2.06; aOR = 1.53, 95% CI 1.25–1.87, respectively). More than once paracetamol use per year and per month were associated with current asthma in a dose–response manner. Additionally, female sex was an independent protective factor for ever asthma (0.82; 0.70–0.96). Among environmental factors, dampness or mildew at home was an independent risk factor for ever asthma (1.50; 1.15–1.97) and current asthma (1.63; 1.21–2.19). Floor heating system was significantly associated with ever asthma (1.57; 1.25–1.98) and current asthma (1.36; 1.04–1.78). Furthermore, dampness or mildew, infrequent house cleaning, and truck traffic in residential streets were significantly associated with asthma symptoms only in old communities, while paracetamol use in the first year of life and flooring materials were significant factors only in new communities.

**Conclusion:**

The prevalence of asthma has increased among preschool children in Shanghai over the past three decades. The identified risk factors indicated the combined effects of genetic, personal, and environmental factors on asthma symptoms. Differentiated strategies should be taken for preventing asthma in old and new communities.

## Introduction

The prevalence of asthma has risen worldwide over the past few decades (1). As the most common chronic respiratory disease in children, it usually starts in early childhood, and half of children with asthma have at least one episode of wheezing in the first 6 years of life (2). The prevalence of asthma in preschool children is higher than that in school-aged children and infants (3), whereas the diagnosis and treatment of asthma in preschool children are challenging due to the lack of objective documentation of reversible airway obstruction and confusion with other diseases such as bronchitis and bronchiolitis. They usually have poorer symptom control and lower quality of life than older children who experience wheezing, putting an increasing burden on families and the healthcare system (4). Thus, it is urgent to fully assess the prevalence and severity of asthma and explore possible risk factors to prevent asthma among preschool children.

The International Study of Asthma and Allergies in Childhood (ISAAC) has carried out the most comprehensive study on the prevalence and associated variables of pediatric asthma using standardized questionnaires throughout the world. According to the ISAAC Phase III study in 2002, the prevalence of childhood asthma among children at the age of 6–7 in China was 6.0%, while it was 4.3% in the 1994 ISAAC Phase I study (5). A nationwide survey in 2010 demonstrated that the prevalence of asthma among children of 0–14 years in Shanghai was 7.6%, which was the highest among the 43 investigated cities in China (3). The prevalence of asthma in China has risen over the last three decades, as dramatic changes have occurred in China's economic and lifestyle. Nevertheless, little is known about recent information on the prevalence and severity in Chinese children since the last ISAAC survey and national survey, especially in children aged 3–6 years. Additionally, the prevalence of asthma in children is rising at a rate that cannot be explained simply by genetic factors. It is a likely complicated and multifactorial condition, which involves combined effects of non-modifiable factors (i.e., genetics and sex) and modifiable factors (i.e., indoor environment and lifestyle) (6). The etiology of asthma remains largely unknown.

Therefore, we conducted the cross-sectional study to explore the prevalence and severity of asthma and identify risk factors for asthma in children at the age of 3–6 years in Shanghai.

## Methods

### Study Design

This is a population-based cross-sectional study carried out in 2021, covering 12 communities and 72 kindergartens in the Yangpu District of Shanghai. Of the 7,096 distributed questionnaires, 6,784 were returned by parents or guardians (response rate: 95.6%). A total of 466 children in day care classes aged 2–3 years were eliminated due to their age, and 135 children were excluded due to partial data loss. Eventually, 6,183 questionnaires were retained as valid.

### Ethics Approval

The Ethics Committee of Shanghai Children's Medical Center, School of Medicine, Shanghai Jiao Tong University approved this study. Informed consent forms were acquired from the parents/caregivers of participants.

### The Questionnaire

Questionnaires consisted of three parts: a core written questionnaire, a personal questionnaire, and an environmental questionnaire, both based on the ISAAC phase III and were translated into Chinese according to the ISAAC translation protocol (7).

In the core written questionnaire, demographic questions on age, sex, school, community, date of birth, height, and weight were asked. The questions used to estimate the prevalence and severity of asthma symptoms were ever asthma, current asthma, 4 or more attacks of wheeze in the past year, severe attack in the past year, wheeze-induced sleep disruption in the last 12 months, wheeze during or after exercise in the past year, and night cough in the past year.

Ever asthma was defined as positive answers to the question “Has your child ever had wheeze or whistle in the chest?” Current asthma was determined based on affirmative answers to the question “Has your child had wheeze or whistle in the chest in the past 12 months?” Physician-diagnosed asthma was identified as parental responses to the question “Has your child ever been diagnosed with asthma by a physician?” Four or more attacks of wheeze and severe attack in the past 12 months were, respectively, determined according to the parental answers to the questions “How many attacks of wheeze has your child had in the last 12 months?” and “has wheeze ever been severe enough to limit your child's speech to only one or two words at a time between breaths?” Wheeze-induced sleep disruption, wheeze during or after exercise, and nocturnal cough in the past 12 months were defined by affirmative responses to the questions “In the past 12 months, has your child had sleep disruption due to wheeze or whistle?” “In the past 12 months, has your child had wheeze or whistle during or after exercise?” and “In the past 12 months, has your child had a dry cough at night in addition to respiratory tract infection?” respectively.

The following variables were analyzed in the personal and environmental questionnaire: paracetamol use in the first year of life, the frequency of paracetamol use in the past year, antibiotic use in the first year of life, smoke exposure at home, pets at home, pets at home in the first year, dampness or mildew, truck traffic in residential streets in the past year, electric heating, floor heating system, electric cooking, gas cooking, solid wood flooring, laminated wood flooring, emulsion paint wall, wallpaper, infrequent house cleaning in the past year, TV viewing in the past year. In addition, genetic factors, including maternal and paternal asthma, rhinitis, and eczema, and the history of personal allergic diseases were collected.

Most of the risk factors mentioned were coded as binary variables by “yes/no” questions, except for the frequency of paracetamol use in the past year (never, more than once per month or more than once per year), heavy truck traffic (never, occasionally, frequently or almost the whole day), infrequent house cleaning in the past year (every day, twice a week, once a week, once every 2 weeks, once a month or less than once a month), and TV viewing in the past year (<1 h per day, 1–3 h per day, 3–5 h per day, or more than 5 h per day).

### Statistical Analysis

Data were coded and analyzed using SPSS 22.0 software. Categorical variables are displayed in frequencies and proportions. Prevalence estimates were computed by the number of affirmative answers to each question divided by the total number of completed answers.

The significance of differences between groups in categorical variables was determined using independent samples chi-squared (χ^2^) test. It was defined as statistically significant that the *p* < 0.05. With 25–30 risk factors potentially associated with asthma, the minimum sample size required approximately 250–300 positive answers to avoid violating the principle of 10 outcome events per variable in the logistic regression. Univariate and multivariate logistic regression analyses were adopted to assess the relationships between the genetic, environmental, and lifestyle variables and ever asthma and current asthma. All significant variables in univariate analyses were subsequently selected to the multivariate logistic regression model. Each regression result was summarized as odds ratio (OR) and corresponding 95% confidence interval (95% CI).

## Results

### The Epidemiology of Asthma Symptoms

There were a total of 6,183 valid questionnaires (3,165 boys and 3,018 girls). The age of the children ranged from 3 to 6 years, with an average of 4.2 ± 0.7 years and a median of 4.1 years. Table 1 shows the positive responses to asthma symptoms. A total of 990 (16.0%) children had ever experienced wheezing, while 695 (11.2%) children had experienced wheezing within the past year. The prevalence of doctor-diagnosed asthma was 5.3% (328/6,183). There were 315 (5.1%) and 906 (14.7%) participants reporting exercise-induced wheezing and night cough in the past year, respectively. Regarding the severity of asthma symptoms, 3.2% of children have experienced sleep disturbance due to wheeze or whistle, while 1.3% of children had no less than 4 attacks of wheeze in the past year.

**Table 1:** Prevalence of asthma in the study (*n* = 6,183).

	**Proportion or prevalence, *n* (%)**	**Male,** ***n* = 3,165**	**Female,** ***n* = 3,018**	***p*-value**
Ever asthma	990 (16.0%)	563 (17.8%)	421 (13.9%)	**<0.001**
Current asthma	695 (11.2%)	391 (12.4%)	304 (10.1%)	**0.005**
Physician-diagnosed asthma	328 (5.3%)	190 (6.0%)	138 (4.6%)	**0.012**
4 or more attacks in the past 12 months	81 (1.3%)	47 (1.5%)	34 (1.1%)	0.217
Severe attack in the past 12 months	24 (0.4%)	11 (0.3%)	13 (0.4%)	0.600
Wheeze-induced sleep disruption in the past 12 months	198 (3.2%)	243 (7.7%)	197 (6.5%)	**<0.001**
Wheeze during or after exercise in the past 12 months	315 (5.1%)	181 (5.7%)	134 (4.4%)	**0.023**
Nocturnal cough in the past 12 months	906 (14.7%)	471 (14.9%)	435 (14.4%)	0.603

*Data are shown as mean (± standard deviation) or number (%). Chi-square test was adopted to assess the significance of between-group differences; p-values of < 0.05 in bold were considered statistically significant*.

The prevalence of asthma symptoms was significantly higher in boys than in girls, but there were no sex differences observed regarding attack frequency and severity or nocturnal cough in the past year.

### Personal and Genetic Factors Associated With Asthma Symptoms by Univariate Analyses

Among the personal and genetic factors shown in Table 2, boys were significantly more likely to have asthma symptoms, and female sex was a significant protective factor for ever asthma (OR = 0.76, 95% CI 0.66–0.87) and current asthma (0.80; 0.68–0.93). The largest OR of personal factors was observed for the association between more than once paracetamol use per month within the past year and current asthma (2.95; 2.10–4.16). More than once paracetamol use per year and per month were both associated with current asthma in a dose–response manner. Strong and significant associations were also seen for antibiotic use in the first year of life (2.19; 1.84–2.60), paracetamol use in the first year of life (1.61; 1.36–1.91), history of allergic rhinitis (2.80; 2.33–3.36), history of eczema (1.90; 1.60–2.26), and history of food allergy (2.34; 1.96–2.80) with current asthma; similarly, significant associations were observed between these factors and ever asthma.

**Table 2:** Personal and parental factors for asthma symptoms by univariate analyses.

**Personal and parental factors**	**Ever asthma**		**Current asthma**	
	**OR (95% CI)**	***p***	**OR (95% CI)**	***p***
Age	1.01 (0.94, 1.09)	0.746	0.94 (0.86, 1.02)	0.151
Community	**1.03 (1.01, 1.05)**	**0.012**	**1.03 (1.01, 1.06)**	**0.014**
Sex				
Male	1.00		1.00	
Female	**0.76 (0.66, 0.87)**	**<0.001**	**0.80 (0.68, 0.93)**	**0.005**
BMI status				
Not overweight or obese	1.00		1.00	
Overweight	1.16 (0.99, 1.37)	0.069	1.08 (0.89, 1.30)	0.455
Obese	1.18 (0.91, 1.53)	0.204	1.13 (0.83, 1.52)	0.440
History of allergic rhinitis	**2.76 (2.35, 3.25)**	**<0.001**	**2.80 (2.33, 3.36)**	**<0.001**
History of eczema	**1.85 (1.59, 2.16)**	**<0.001**	**1.90 (1.60, 2.26)**	**<0.001**
History of food allergy	**2.14 (1.83, 2.50)**	**<0.001**	**2.34 (1.96, 2.80)**	**<0.001**
Antibiotic use (1st year)	**2.27 (1.95, 2.64)**	**0.017**	**2.19 (1.84, 2.60)**	**<0.001**
Paracetamol use (1st year)	**1.62 (1.40, 1.88)**	**<0.001**	**1.61 (1.36, 1.91)**	**<0.001**
Frequency of paracetamol use				
Never	1.00		1.00	
>Once/year	**1.54 (1.26, 1.89)**	**<0.001**	**1.71 (1.33, 2.19)**	**<0.001**
>Once/month	**2.22 (1.65, 3.01)**	**<0.001**	**2.95 (2.10, 4.16)**	**<0.001**
Watching TV				
<1 h/day	1.00		1.00	
1–3 h/day	1.04 (0.90, 1.20)	0.611	1.04 (0.88, 1.23)	0.662
3–5 h/day	0.99 (0.60, 1.60)	0.957	0.96 (0.54, 1.72)	0.887
>5 h/day	Number too small		Number too small	
Maternal rhinitis	**3.06 (2.47, 3.78)**	**<0.001**	**2.95 (2.34, 3.73)**	**<0.001**
Maternal asthma	**5.40 (3.58, 8.13)**	**<0.001**	**5.47 (3.59, 8.34)**	**<0.001**
Maternal eczema	**2.03 (1.46, 2.83)**	**<0.001**	**2.12 (1.47, 3.05)**	**<0.001**
Paternal rhinitis	**2.89 (2.36, 3.53)**	**<0.001**	**2.73 (2.18, 3.14)**	**<0.001**
Paternal asthma	**5.90 (4.04, 8.60)**	**<0.001**	**7.19 (4.92, 10.53)**	**<0.001**
Paternal eczema	1.21 (0.80, 1.83)	0.372	1.28 (0.80, 2.04)	0.301

*OR, odds ratio; CI, confidence interval; 1st year, in the first year of life. Bold font means statistical significance (p < 0.05)*.

Both maternal and paternal rhinitis, asthma, and eczema were significantly associated with ever asthma and current asthma. Among the hereditary factors shown in Table 2, the strongest association was seen between paternal asthma and current asthma (7.19; 4.92–10.53). Maternal asthma had the second strongest association with current asthma (5.47; 3.59–8.34).

### Environmental Factors Associated With Asthma Symptoms by Univariate Analyses

Among environmental factors shown in Table 3, dampness at home had the strongest associations with ever asthma (1.90; 1.51–2.40) and current asthma (2.10; 1.62–2.71). Both pets at home in the first year of life and pets at home in the past year were associated with current asthma, with ORs of (1.33, 1.09–1.63) and (1.42; 1.14–1.78), respectively. Additionally, smoke exposure at home was associated with a significantly increased risk of ever asthma (1.19; 1.02–1.39) and current asthma (1.24; 1.04–1.47). Cleaning the house once a week and once a month were significantly associated with the increased risk of current asthma with ORs of (1.26; 1.02–1.56) and (1.90; 1.16–3.12), respectively. Occasional, frequent, and almost constant truck traffic were significantly associated with the increased risk of current asthma with ORs of (1.24; 1.01–1.52), (1.75; 1.31–2.33), and (1.59; 1.07–2.38), respectively. Using the floor heating system showed significant associations with ever asthma 1.42 (1.16, 1.74) and current asthma (1.32; 1.04–1.68). Solid wood flooring turned out to be a protective factor for ever asthma (0.86; 0.75–1.00), while laminated flooring was a significant risk factor (1.23; 1.06–1.44). The use of emulsion paint was significantly associated with ever asthma (1.18; 1.03–1.35).

**Table 3:** Environmental factors for asthma symptoms by univariate analyses.

**Environmental factors**	**Ever asthma**		**Current asthma**	
	**OR (95% CI)**	***p***	**OR (95% CI)**	***p***
Smoking exposure at home	**1.19 (1.02, 1.39)**	**0.024**	**1.24 (1.04, 1.47)**	**0.018**
Pets at home	**1.33 (1.12, 1.58)**	**0.001**	**1.33 (1.09, 1.63)**	**0.005**
Pets at home (1st year)	**1.32 (1.08, 1.60)**	**0.006**	**1.42 (1.14, 1.78)**	**0.002**
Dampness or mildew	**1.90 (1.51, 2.40)**	**<0.001**	**2.10 (1.62, 2.71)**	**<0.001**
Infrequent house cleaning				
Everyday	1.00		1.00	
Twice/week	1.16 (0.98, 1.39)	0.089	1.10 (0.89, 1.35)	0.368
Once/week	**1.31 (1.09, 1.57)**	**0.004**	**1.26 (1.02, 1.56)**	**0.032**
Once/two week	**1.59 (1.09, 2.32)**	**0.015**	1.46 (0.95, 2.27)	0.087
Once/month	1.44 (0.90, 2.32)	0.133	**1.90 (1.16, 3.12)**	**0.011**
< Once/month	1.43 (0.80, 2.53)	0.224	1.46 (0.76, 2.78)	0.256
Truck traffic in residential streets				
Never	1.00		1.00	
Occasionally	**1.43 (1.19, 1.72)**	**<0.001**	**1.24 (1.01, 1.52)**	**0.044**
Frequently	**1.85 (1.43, 2.39)**	**<0.001**	**1.75 (1.31, 2.33)**	**<0.001**
Almost always	**1.62 (1.13, 2.32)**	**0.008**	**1.59 (1.07, 2.38)**	**0.023**
Heating				
Electric heating	0.92 (0.78, 1.09)	0.330	0.96 (0.80, 1.17)	0.707
Floor heating system	**1.42 (1.16, 1.74)**	**0.001**	**1.32 (1.04, 1.68)**	**0.022**
Cooking				
Electric cooking	1.15 (0.97, 1.36)	0.119	1.05 (0.86, 1.29)	0.633
Gas cooking	0.93 (0.76, 1.14)	0.486	0.96 (0.76, 1.21)	0.722
Floor				
Solid wood flooring	**0.86 (0.75, 1.00)**	**0.045**	0.89 (0.75, 1.05)	0.176
Laminated flooring	**1.23(1.06, 1.44)**	**0.007**	1.17 (0.98, 1.40)	0.077
Wall				
Emulsion paint	**1.18 (1.03, 1.35)**	**0.018**	1.04 (0.89, 1,22)	0.610
Wallpaper	0.99 (0.84, 1.16)	0.877	1.04 (0.86, 1.25)	0.710

*OR, odds ratio; CI, confidence interval; 1st year, in the first year of life. Bold font means statistical significance (p < 0.05)*.

### Adjusted Risk Factors for Asthma Symptoms

Table 4 presents adjusted risk factors for asthma symptoms analyzed by multivariate logistic regression analyses. The associations for both maternal and paternal rhinitis and asthma were maintained, although evidences of differences slightly decreased, and parental eczema was found to be a non-significant factor. The strongest associations were likewise between paternal asthma and ever asthma (4.89; 3.23–7.40) and current asthma (5.91; 3.87–9.01). Female sex had a significant protective effect, and the association between female sex and ever asthma was slightly weaker after adjusting for confounders (0.82; 0.70–0.96). Furthermore, there were significant associations between history of allergic rhinitis (1.89; 1.52–2.34), history of eczema (1.34; 1.09–1.64), history of food allergy (1.68; 1.37–2.06), antibiotic use in the first year of life (1.53; 1.25–1.87), and current asthma. These factors were significantly associated with ever asthma as well. Interestingly, a dose–response effect was observed between the frequency of paracetamol use and asthma symptoms. The aOR of more than once paracetamol use per year with current asthma was 1.29, while the aOR of more than once paracetamol use per month with current asthma was 2.06. The effect was similarly seen between paracetamol use within the past year and ever asthma. Among environmental factors, dampness in the house remained significantly associated with ever asthma (1.50; 1.15–1.97) and current asthma (1.63; 1.21–2.19). Floor heating system was significantly associated with ever asthma (1.57; 1.25–1.98) and current asthma (1.36; 1.04–1.78). In contrast, smoke exposure at home, pets at home, and heavy truck traffic lost significance in the multivariate model.

**Table 4:** Adjusted associations between risk factors and asthma symptoms by multivariate analyses.

**Factors**	**Ever asthma**		**Current asthma**	
	**aOR (95% CI)**	***p***	**aOR (95% CI)**	***p***
Community	**1.03 (1.01, 1.06)**	**0.008**	**1.03 (1.01, 1.06)**	**0.019**
Sex				
Male	1.00		1.00	
Female	**0.82 (0.70, 0.96)**	**0.012**	0.85 (0.71, 1.01)	0.069
History of allergic rhinitis	**1.86 (1.53, 2.25)**	**<0.001**	**1.89 (1.52, 2.34)**	**<0.001**
History of eczema	**1.30 (1.09, 1.56)**	**0.004**	**1.34 (1.09, 1.64)**	**0.005**
History of food allergy	**1.57 (1.31, 1.89)**	**<0.001**	**1.68 (1.37, 2.06)**	**<0.001**
Antibiotics use (1st year)	**1.68 (1.41, 2.00)**	**<0.001**	**1.53 (1.25, 1.87)**	**<0.001**
Paracetamol use (1st year)	**1.26 (1.06, 1.51)**	**0.010**	1.21 (0.99,1.48)	0.060
Frequency of paracetamol use				
Never	1.00		1.00	
>Once/year	1.16 (0.91, 1.47)	0.223	1.29 (0.97, 1.71)	0.077
>Once/month	**1.44 (1.01, 2.04)**	**0.043**	**2.06 (1.39, 3.04)**	**<0.001**
Maternal rhinitis	**2.18 (1.71, 2.78)**	**<0.001**	**1.94 (1.48, 2.54)**	**<0.001**
Maternal asthma	**4.06 (2.57, 6.41)**	**<0.001**	**3.85 (2.40, 6.17)**	**<0.001**
Maternal eczema	1.37 (0.94, 2.00)	0.104	1.42 (0.93, 2.16)	0.104
Paternal rhinitis	**1.76 (1.39, 2.22)**	**<0.001**	**1.67 (1.29, 2.16)**	**<0.001**
Paternal asthma	**4.89 (3.23, 7.40)**	**<0.001**	**5.91 (3.87, 9.01)**	**<0.001**
Smoking exposure at home	1.05 (0.88, 1.25)	0.558	1.10 (0.91, 1.35)	0.328
Pets at home	1.12 (0.88, 1.41)	0.358	1.09 (0.83, 1.42)	0.550
Pets at home (1st year)	1.07 (0.82, 1.40)	0.614	1.13 (0.83, 1.52)	0.438
Dampness or mildew	**1.50 (1.15, 1.97)**	**0.003**	**1.63 (1.21, 2.19)**	**0.001**
Cleaning house				
Everyday	1.00		1.00	
Twice/week	**1.24 (1.02, 1.51)**	**0.031**	1.12 (0.89, 1.40)	0.334
Once/week	**1.34 (1.09, 1.64)**	**0.006**	1.24 (0.98, 1.57)	0.080
Once/two week	**1.59 (1.04, 2.43)**	**0.031**	1.49 (0.92, 2.41)	0.108
Once/month	1.24 (0.70, 2.20)	0.458	1.62 (0.90, 2.91)	0.111
< Once/month	1.36 (0.70, 2.63)	0.362	1.20 (0.56, 2.55)	0.641
Truck traffic in home's street				
Never	1.00		1.00	
Occasionally	1.15 (0.93, 1.41)	0.193	0.98 (0.77, 1.24)	0.862
Frequently	1.17 (0.87, 1.58)	0.304	1.10 (0.79, 1.53)	0.582
Almost always	1.32 (0.89, 1.96)	0.172	1.22 (0.78, 1.90)	0.388
Floor heating system	**1.57 (1.25, 1.98)**	**<0.001**	**1.36 (1.04, 1.78)**	**0.025**
Floor				
Solid wood	0.98 (0.71, 1.35)	0.900	0.94 (0.66, 1.35)	0.741
Laminated flooring	1.06 (0.75, 1.49)	0.730	0.94 (0.64, 1.39)	0.762
Emulsion paint	1.14 (0.98, 1.33)	0.098	1.00 (0.84, 1.20)	0.999

*aOR, adjusted odds ratio; CI, confidence interval; 1st year, in the first year of life. Odds ratios shown in the table were adjusted for all other risk/protective factors listed by multivariate logistic regression. Bold font means statistical significance (p < 0.05)*.

### The Prevalence and Risk Factors for Asthma Symptoms Stratified by Community

**Figure 1:**
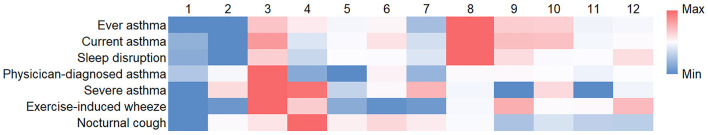
The prevalence of asthma symptoms stratified by community. The heatmap displays the differential prevalence of asthma symptoms across the communities. Blue represents low value, and red represents high value. (1) Daqiao community, (2) Dinghai community, (3) Jiangpu community, (4) Kongjiang community, (5) Pingliang community, (6) Siping community, (7) Wujiaochang community, (8) Xinjiangwan community, (9) Yanji community, (10) Yinhang community, (11) Changbai community, and (12) Changhai community.

Previous studies have reported the differences in the prevalence of allergic diseases between urban and rural areas. The Yangpu district in our study is a characteristic one in which old communities and modern areas exist. The above data in Table 4 showed that community was an independent risk factor for asthma symptoms; to be specific, significant associations between community and ever asthma (*P* = 0.008) and current asthma (*P* = 0.019) were observed from the data of multivariate logistic regression analyses, indicating that the prevalence of asthma symptoms varied between communities. Consequently, we assessed the prevalence of asthma symptoms stratified by community (Figure 1). In general, the prevalence of asthma symptoms in the Xinjiangwan community and the Jiangpu community was higher than that in other communities, especially than that in the Daqiao community, Dinghai community, and Wujiaochang community. For instance, the prevalence of ever asthma in the Daqiao community, Dinghai community, Wujiaochang community, Xinjiangwan community, and Jiangpu community was 10.6, 10.6, 13.2, 20.2, and 17.7%, respectively, while the prevalence of current asthma was 7.5, 5.6, 9.7, 14.5 and 13.4%, respectively (specific data can be found in the Supplementary Materials). Interestingly, the Xinjiangwan community and the Jiangpu community, which had a higher prevalence of asthma symptoms, are more modern and developed areas in the Yangpu district, while the Daqiao community, the Dinghai community, and the Wujiaochang community are older and less developed communities. Thus, these communities were selected as representative of new and old communities.

Table 5 shows the ORs and 95% CIs of significant risk factors for current asthma in old and new communities analyzed by univariate logistic regression. Considering that the ORs of genetic factors in old communities were higher than those in new communities, the associations between genetic factors and current asthma were stronger in old communities. The associations of dampness at home (2.95; 1.72–5.08), infrequent house cleaning (1.20; 1.04–1.39), and truck traffic in residential streets (1.32; 1.03–1.68) with current asthma were significant only in old communities; in contrast, the significant associations of solid wood flooring (0.68; 0.47–0.98) and laminated flooring (1.54; 1.06–2.26) with current asthma were only observed in new communities.

**Table 5:** The significant risk factors for current asthma in old and new communities analyzed by univariate analyses.

**Factors**	**Old communities**		**New communities**	
	**OR (95% CI)**	***p***	**OR (95% CI)**	***p***
History of allergic rhinitis	**3.14 (2.01, 4.90)**	**<0.001**	**2.19 (1.46, 3.30)**	**<0.001**
History of eczema	**1.68 (1.09, 2.60)**	**0.020**	**2.40 (1.67, 3.46)**	**<0.001**
History of food allergy	**3.14 (2.04, 4.84)**	**<0.001**	**2.33 (1.59, 3.42)**	**<0.001**
Antibiotic use (1st year)	**1.87 (1.20, 2.90)**	**0.005**	**2.30 (1.58, 3.34)**	**<0.001**
Paracetamol use (1st year)	1.41 (0.92, 2.15)	0.119	**2.04 (1.41, 2.95)**	**<0.001**
Frequency of paracetamol use	**1.79 (1.18, 2.72)**	**0.006**	**1.58 (1.07, 2.33)**	**0.022**
Maternal rhinitis	**3.49 (1.93, 6.31)**	**<0.001**	**2.49 (1.51, 4.13)**	**<0.001**
Maternal asthma	**6.56 (2.16, 19.92)**	**0.001**	**5.87 (2.34, 14.70)**	**<0.001**
Paternal rhinitis	**3.45 (2.03, 5.83)**	**<0.001**	**2.08 (1.27, 3.41)**	**0.004**
Paternal asthma	**8.05 (3.67, 17.65)**	**<0.001**	**6.69 (3.04, 14.74)**	**<0.001**
Dampness or mildew	**2.95 (1.72, 5.08)**	**<0.001**	1.57 (0.88, 2.80)	0.123
Infrequent house cleaning	**1.20 (1.04, 1.39)**	**0.016**	1.05 (0.89, 1.24)	0.536
Truck traffic in residential streets	**1.32 (1.03, 1.68)**	**0.028**	1.25 (0.98, 1,59)	0.068
Floor				
Solid wood flooring	0.83 (0.56, 1.25)	0.378	**0.68 (0.47, 0.98)**	**0.039**
Laminated flooring	1.34 (0.87, 2.06)	0.190	**1.54 (1.06, 2.26)**	**0.025**

*OR, odds ratio; CI, confidence interval; 1st year, in the first year of life. Bold font means statistical significance (p < 0.05)*.

## Discussion

Our study found that the prevalence of ever asthma and current asthma in Shanghai (Southeastern China) was 16.0 and 11.2%, respectively, 2.4-fold and 3.4-fold higher than those in Shanghai during the 1994 ISAAC I study (8). According to the ISAAC III study in 2002, there were regional differences in the prevalence of current asthma in children aged 6–7 (Indian subcontinent: 6.8%, Asia-Pacific: 9.5%, Western Europe: 9.6%, Africa: 10%, Latin America: 17.3%) (9). A study in 2007 found that the prevalence of ever asthma and current asthma among children aged 2–6 years in mainland China was 8.1 and 3.4%, respectively (10). Our data showed that the prevalence of asthma has increased among preschool children in Shanghai over the past three decades. Furthermore, heterogeneity was observed in the prevalence of asthma among 12 communities. The highest prevalence of lifetime asthma and current asthma was seen in the Xinjiangwan community (20.2% and 14.5%), 1.9- and 2.6-fold higher than that in the Dinghai community, which had the lowest prevalence.

Interestingly, a higher incidence appeared to occur in more developed and newer communities, in accordance with the points that industrialization increased the risk of allergic disease, and children in urban areas were more prone to allergic-related diseases (11). Therefore, we classified the population by community, and logistic regression analyses revealed that, among environmental factors, solid wood flooring was a protective factor for current asthma and laminated flooring was a risk factor only in new and developed communities. One possible explanation is that the houses in new communities are more likely to be decorated recently and contain new furniture, which could aggravate indoor air pollution. Huang et al. revealed that indoor painting and purchasing new furniture could increase the concentration of volatile chemicals in the house, especially formaldehyde (12). Rumchev et al. reported that domestic exposure to formaldehyde increased the risk of childhood asthma (13). In contrast, our data showed dampness at home, cleaning the house infrequently, and heavy truck traffic only significantly increased the risk of asthma symptoms in old communities, which was consistent with a cross-sectional study in Turkey conducted by Kurt and colleagues. They found that dampness and visible molds increased the risk of allergic diseases, especially in rural areas (14). In addition, the reason why the effect of heavy traffic on asthma symptoms was only observed in old communities could be that new communities often have better infrastructure with underground parking garages in Shanghai, while parking areas in old communities are usually on the ground, which could cause more truck traffic. These results indicated that children living in different types of communities had differentiated patterns of risk factors, and differential interventions should be taken to prevent asthma.

In our study, boys experienced a higher prevalence of asthma symptoms in both univariate and multivariate logistic regression analyses. Similar results have been found in other studies (15). A potential explanation is the anatomical differences between boys and girls. Interestingly, the trend changes along with age. The incidence of asthma in boys did not increase during puberty and eventually declined at the end of adolescence, while the prevalence and severity in girls began rising at the age of 9–10 (16, 17). Sex hormones may play a crucial role in this change. In our multivariate logistic regression analyses, paracetamol use was an independent risk factor, whether in the first year of life or the last year, and this significant association was observed in a dose-dependent manner. In addition, antibiotics use during infancy was significantly associated with asthma. The first year of life is a critical period for the development of a child's immune system, and the gut microbiota plays a vital role in this process; however, antibiotic intake may lead to the disturbance of intestinal flora, which can cause an imbalance of the immune system and result in the development of asthma (18). Accordingly, the unnecessary and irrational prescription of paracetamol and antibiotics should be reduced.

Genetic risk factors play a more crucial role in childhood-onset asthma than in adult-onset asthma (19). Therefore, our study comprehensively analyzed the effect of parental allergic history on childhood asthma symptoms and found that both maternal and paternal atopy, including asthma and rhinitis, increased the risk of childhood asthma in both univariate and multivariate logistic regression analyses, which could be explained by *in utero* exposures, hormone differences, and genetic imprinting (20, 21). A meta-analysis found that the OR for asthma in children whose mothers had asthma compared to mothers without asthma was 3.0, while the corresponding OR for asthma in children of asthmatic fathers was 2.4 (22). Lim et al. reported that inflammatory cytokines could be transmitted through the placenta, modifying the fetal immune system (23). Children receiving breast milk with higher levels of leptin and insulin had an increased risk of developing asthma before the age of 3 years (24). However, in our study, a stronger association was observed between paternal asthma and current asthma (5.91; 3.87–9.01), compared to maternal asthma (3.85; 2.40–6.17), which is consistent with previous studies (25–28). The maternal effect on childhood asthma may be caused by genetic or environmental factors shared *in utero*, while the paternal effect on childhood asthma is more likely due to genetics (29). An Australian study demonstrated that a region on chromosome 7p was tightly associated with asthma, and the linkage was paternally derived (30).

In the previous studies, the association between environmental factors and asthma has been less consistent (6). In the present study, dampness or mildew at home was an independent risk factor, with aORs of 1.50 and 1.63 for ever asthma and current asthma, respectively, in line with previous studies in recent years (31, 32). A recent case–control study in Uganda has demonstrated that schoolchildren who lived in areas with frequent truck traffic were 2.59-fold more likely to have asthma symptoms (33). It is generally accepted that tobacco smoke and particle pollution facilitate allergic sensitization to common allergens and increase the tendency of allergy (34). Smoking exposure and truck traffic were significant factors in our univariate analyses, but failed to be significant in the multivariate analyses. The significance was lost, probably because we did not stratify the factor according to the level and period of smoke exposure. A previous study demonstrated that household smoke exposure increased the risk for asthma symptoms among primary school students in a dose–response manner (35). The level of smoke exposure was closely associated with asthma outcomes. It was reported that only heavy maternal smoking (more than 20 cigarettes per day) during pregnancy was significantly associated with the increased risk of adolescent asthma symptoms (36). Besides, the effects of smoke exposure in different periods were explored in previous studies. A birth cohort study over a 3-year period revealed that prenatal maternal smoking significantly increased the risk for wheezing illness with the incidence rate ratio of 2.09, while the incidence rate ratio of postnatal maternal smoking was 1.27 (37). A recent meta-analysis also showed that prenatal smoke exposure had a greater negative effect on childhood asthma than postnatal exposure (38). Accordingly, failure to stratify smoke exposure by extent and period may contribute to the insignificant difference in our multivariate analyses.

The present study has several strengths and limitations. Firstly, this was a cross-sectional study, and the investigation of casual connections between risk factors and asthma was limited. The questionnaires may be subject to recall bias. However, cross-sectional studies are ideally suited for assessing the prevalence of asthma, which was a major concern of this study. Besides, the ISAAC questionnaire used in this study has been extensively validated in different countries. Secondly, only one district was included in our study, and this single-center nature may weaken the representativeness of our data. However, the Yangpu district is a characteristic district that contains old communities and modern areas, so we classified the population by community and explored the differences in risk factors for asthma in old and new communities. Moreover, the response rate was excellent, and thus the reported prevalence was comprehensive.

Overall, the present cross-sectional epidemiological study found that the prevalence of asthma symptoms among children aged 3–6 years has increased in Shanghai during the past three decades. The identified risk and protective factors suggested that genetic, personal, and environmental factors had a combined effect on asthma symptoms among preschool children. Among modifiable factors, antibiotic use, and paracetamol use in the first year of life, current paracetamol use, and dampness at home were independent risk factors for asthma. Additionally, the patterns of risk factors for asthma were different between old and new communities; consequently, differentiated strategies should be taken to help families and healthcare specialists prevent asthma and its attacks.

## Data Availability Statement

The original data of this study are available from the corresponding author, upon reasonable request.

## Author Contributions

YB designed the study, drafted the original version, and confirmed the final manuscript when submitted. JR, JX, and PZ conducted the survey and collected data. JR and PZ performed data disposal and analyses. JR completed the original manuscript. All authors have reviewed and corrected the manuscript, approved the submission of the final manuscript, and take responsibility for the content of this study.

## Funding

This work was supported by the National Natural Science Foundation of China (Grant Number: 81870024). The funders were not involved in any process of this study.

## Conflict of Interest

The authors declare that the research was conducted in the absence of any commercial or financial relationships that could be construed as a potential conflict of interest.

## Publisher's Note

All claims expressed in this article are solely those of the authors and do not necessarily represent those of their affiliated organizations, or those of the publisher, the editors and the reviewers. Any product that may be evaluated in this article, or claim that may be made by its manufacturer, is not guaranteed or endorsed by the publisher.
