# Supplementary material for: Prevalence and Risk Factors of Asthma in Preschool Children in Shanghai, China: A Cross-Sectional Study
Source: Front Pediatr. 2022 Feb 9;9:793452. doi: 10.3389/fped.2021.793452 (PMC8864107; doi:10.3389/fped.2021.793452)
Supplement: Supplementary file 1 [file Table_1.DOCX]

Supplementary Material

Table 1 The prevalence of asthma symptoms stratified by community

|  | Community | | | | | | | | | | | |
| --- | --- | --- | --- | --- | --- | --- | --- | --- | --- | --- | --- | --- |
|  | 1 | 2 | 3 | 4 | 5 | 6 | 7 | 8 | 9 | 10 | 11 | 12 |
| Ever wheeze | 10.6% | 10.6% | 17.7% | 16.7% | 16.0% | 16.4% | 13.2% | 20.2% | 17.5% | 17.4% | 15.8% | 16.0% |
| Current asthma | 7.5% | 5.6% | 13.4% | 10.0% | 11.1% | 11.7% | 9.7% | 14.5% | 12.6% | 12.5% | 10.9% | 11.2% |
| Sleep disruption | 4.4% | 3.2% | 8.2% | 5.8% | 7.1% | 7.0% | 6.3% | 10.7% | 7.9% | 7.0% | 7.1% | 7.8% |
| Physician-diagnosed asthma | 4.6% | 5.6% | 15.3% | 4.0% | 3.3% | 6.3% | 4.2% | 5.9% | 5.9% | 5.7% | 5.4% | 5.8% |
| Severe asthma | 0.0% | 0.5% | 0.9% | 0.8% | 0.2% | 0.4% | 0.6% | 0.3% | 0.0% | 0.5% | 0.0% | 0.3% |
| Exercise-induced wheeze | 3.5% | 3.7% | 8.0% | 6.1% | 4.0% | 3.6% | 3.7% | 5.0% | 6.7% | 5.1% | 5.2% | 6.4% |
| Nocturnal cough | 10.4% | 15.3% | 15.8% | 18.6% | 15.5% | 16.1% | 15.6% | 15.1% | 12.8% | 14.2% | 13.4% | 13.3% |

Data were showed as number (%). 1.Daqiao community 2.Dinghai community 3.Jiangpu community 4.Kongjiang community 5.Pingliang community 6.Siping community 7.Wujiaochang community 8.Xinjiangwan community 9.Yanji community 10.Yinhang community 11.Changbai community 12.Changhai community
