# Supplementary material for: Prevalence and Risk Factors of Asthma in Preschool Children in Shanghai, China: A Cross-Sectional Study
Source: Front Pediatr. 2022 Feb 9;9:793452. doi: 10.3389/fped.2021.793452 (PMC8864107; doi:10.3389/fped.2021.793452)
Supplement: Supplementary file 2 [file Data_Sheet_1.PDF]

QUESTIONNAIRE

We thank you sincerely for participating in this study. The questionnaire is about general sociodemographic and health related questions. The information you fill out is very important for this study. Therefore, we politely ask you to read questions carefully and fill them out truthfully.

On this sheet are questions about your child's name, age, gender, ethnicity, weight, height, school, community, contact details, and birth dates. Please write your answers to these questions in the space provided.
All other questions require you to tick your answer in a box. If you make a mistake, put a cross in the box and tick the correct answer. Tick only one option unless otherwise instructed.

SCHOOL: ☐

COMMUNITY: ☐

TODAY'S DATE: ☐ Day ☐ Month ☐ Year

CHILD'S NAME: ☐

CHILD'S AGE: ☐ years

CHILD'S DATE OF BIRTH: ☐ Day ☐ Month ☐ Year

CHILD'S HEIGHT: ☐ CM

CHILD'S WEIGHT: ☐ KG

CHILD'S ETHNICITY: ☐

CONTACT DETAILS: ☐

(Tick all your answers for the rest of the questionnaire)

Is your child a: BOY ☐ GIRL ☐

Module 1 Core questionnaire for wheezing and asthma

1 Has your child ever had wheezing or whistling in the chest at any time in the past?

Yes

No

IF YOU HAVE ANSWERED "NO" PLEASE SKIP TO QUESTION 6

2 Has your child had wheezing or whistling in the chest in the last 12 months?

Yes ☐

No ☐

IF YOU HAVE ANSWERED "NO" PLEASE SKIP TO QUESTION 6

---

3 How many attacks of wheezing has your child had in the last 12 months?

None ☐

1 to 3 ☐

4 to 12 ☐

More than 12 ☐

4 In the last 12 months, how often, on average, has your child's sleep been disturbed due to wheezing?

Never woken with wheezing ☐

Less than one night per week ☐

One or more nights per week ☐

5 In the last 12 months, has wheezing ever been severe enough to limit your child's speech to only one or two words at a time between breaths?

Yes ☐

No ☐

---

6 Has your child ever had asthma?

Yes ☐

No ☐

7 In the last 12 months, has your child's chest sounded wheezy during or after exercise?

Yes ☐

No ☐

8 In the last 12 months, has your child had a dry cough at night, apart from a cough associated with a cold or chest infection?

Yes ☐

No ☐

Module 2 Questionnaire about personal and genetic factors

1. In the first 12 months of your child's life, did you usually give paracetamol for fever?

Yes ☐

No ☐

2. In the past 12 months, how often, on average, have you given your child paracetamol?

Never ☐

At least once a year ☐

At least once per month ☐

3. In the first 12 months of life, did your child have any antibiotics?

Yes ☐

No ☐

4. Did your child has eczema, urticaria, diarrhea, lips or eyes swelling, cough or asthma and other allergic symptoms due to food allergy?

Yes ☐

No ☐

If "yes", what kind of food are your child allergic to? ☐

5. Has your child ever been diagnosed with the following allergic diseases by a physician? (You may choose more than one options)

asthma ☐

rhinitis ☐

eczema ☐

6. Does any of your family members have allergic history?

Yes ☐

No ☐

If “yes”, what is the problem with which family member? ☐ ☐

Module 3 Questionnaire about lifestyle and environmental factors

1. Did you have a pet in your home during the first year of your child’s life?

Yes ☐

No ☐

If “yes”, what kind of pet did you have? ☐

2. In the past 12 months, have you had a pet in your home?

Yes ☐

No ☐

If “yes”, what kind of pet did you have? ☐

3. How often do you clean the room where your child sleep?

Everyday ☐

Twice per week ☐

Once per week ☐

Once every two weeks ☐

Once per month ☐

Less than once a month ☐

4. During a normal week, how many hours a day does your child watch television?

- Less than 1 hour ☐
- 1 hour but less than 3 hours ☐
- 3 hours but less than 5 hours ☐
- 5 hours or more ☐

5. Does any family members who live together smoke?

- Yes ☐
- No ☐

If “yes”, which family members smoke? ☐

6. How often do trucks pass through the street where you live?

- Never ☐
- Seldom ☐
- Frequently through the day ☐
- Almost the whole day ☐

7. In your house, what fuel is usually used for cooking? (You may choose more than one options)

- Electricity ☐
- Gas ☐
- Wood ☐
- Other - please specify ☐

8. In your house, what fuel is usually used for heating? (You may choose more than one options)

- Electricity ☐
- Floor heating system ☐
- Gas ☐
- Other - please specify ☐

–

9. What material is the floor of room where your child sleeps? (You may choose more than one options)

- Solid wood ☐
- Laminated floor ☐
- Other - please specify ☐

10. What material is the wall of room where your child sleeps? (You may choose more than one options)

- Emulsion paint ☐
- Wallpaper ☐
- Other - please specify ☐

11. Is the room where your child sleeps damp or moldy?

- Yes ☐
- No ☐

Thank you very much for your help with this questionnaire. We sincerely appreciate your assistance.
